# Diabetes Insipidus

**Published:** 1909-03-13

**Authors:** 


### Medicine.

### DIABETES INSIPIDUS.

The following cases illustrate many points about diabetes insipidus, amongst them being:—(1) The fact that the polyuria is primary; that is to say, that the patient drinks to replace that which he cannot help losing by his kidneys, and does not micturate abundantly merely to get rid of what he drinks in excess; (2) that the disease may arise without any apparent cause; (3) that, on the other hand, it may be attributable to some definite cause such as head injury or fright; (4) that in some instances there is a hereditary factor in the occurrence of the malady; (5) that life is not necessarily in danger on account of the disease; (6) that death results from intercurrent affections, such as phthisis or pneumonia, and not from the supervention of glycosuria and coma; (7) that the disease may originate during pregnancy; (8) that it may occur even in quite young children.

*Case* 1.—A boy, aged four, of nervous, changeable temperament and considerable precocity, had been quite well till he was two years old. He then suffered from an attack of summer diarrhœa and vomiting. The gastro-intestinal symptoms were soon cured, but the child did not recover perfect health. About this time the mother first noticed that he was micturating both frequently and abundantly and that he was constantly wanting to drink. These symptoms steadily increased; whether they were really augmented by the intercurrent measles and other childish ailments he suffered from and survived it is difficult to say; the symptoms steadily became more obvious and troublesome, though the patient recovered from the other maladies that attacked him from time to time.

When seen at four years of age the most striking point about him was the extraordinary keenness he evinced for fluids of every sort. He was not a drunkard in the sense that he desired alcohol. Drink he must, but water sufficed him. He was obliged to drink, and nothing could keep him from it. The mother had tried to stop it by withholding fluids, but the child's mouth and lips rapidly became parched. As frequently happens in these cases the child was wont to drink the contents of the bedroom ewer in the night time. Upon the average, though he was only four years old, he used to drink between one and a half and two gallons per diem. Appetite, on the other hand, was capricious and it was often difficult to get the boy to eat anything at all.

The urine voided in each twenty-four hours averaged over a gallon and a half. It was almost colourless, of specific gravity 1006, and feebly alkaline reaction; analysis showed that it contained the normal constituents of urine in very dilute solution, and no abnormal substances—in other words it was typical of diabetes insipidus.

*Case* 2.—This also concerns a male child, aged four, but differs from Case 1, first in being less severe, and, secondly, in being hereditary.

It seems to be particularly interesting in that, to judge by the course the malady followed in mother and grandmother, it is likely that the condition will cease to trouble the patient when adult life is reached. Born at term, the infant was breast fed. At five months a little soup was given from time to time as well as the breast; weaning did not occur until the unusually late time of twenty-one months. Up to this time nothing wrong had been noticed; the child neither ate nor drank more than an average child of the same age does.

A month later, that is to say when twenty-two months old, he was noticed to be always thirsty. At the sight of water he would cry out, and when it was offered to him he would seize upon the glass or cup and drink its contents with avidity. This thirst steadily increased; everything of a fluid nature that came to the child's hand was immediately consumed—water, wine, soapsuds, and even the water of urinals. He had been seen to drink nearly a quart of water straight off. Naturally he had a dilated stomach. Bodily he was strong, but with a decided tendency to rickets. The urine passed averaged about 100 oz. daily; it contained neither sugar nor albumen, and it was, in fact, a very dilute normal urine.

It is interesting to note that the mother remembers that she suffered from precisely similar polydipsia and polyuria when she was a girl, and so did the maternal grandmother, though in both their cases the symptoms entirely abated when adult life was reached. There was nothing of the kind on the father's side of the house. A similar case of hereditary diabetes insipidus has recently been quoted in the discussion upon Heredity before the Royal Society of Medicine.

*Case* 3.—A man, of middle age, was in perfect health so far as he knew until he met with a severe bicycling accident. He was thrown over the handlebars of his machine, but does not know what else happened. He was found unconscious, and was taken home. He remained completely comatose for a fortnight, during which time cerebro-spinal fluid dripped abundantly from his nose. It seemed clear that he had fractured the anterior part of the base of his skull, and this was confirmed later by the development of bilateral temporal hemianopia, which was presumably due to callus interfering with the decussating fibres of his optic chiasma. He recovered consciousness at the end of a fortnight, and his first sensation was that of extreme thirst. He became perfectly well in the course of time, except for the persistence of his visual impairment upon the one hand, and of his polydipsia and polyuria upon the other. He could not pass a single night without rising several times both to drink abundance of water and to pass abundance of clear pale urine of specific gravity 1006, free from sugar and from albumin.

This patient used to pass an average of about ten pints of urine in 24 hours, and he used to drink about one-third more than this. Upon one occasion he tried to see if glycosuria could be induced more readily in him than is the case in other people, but he did not develop glycosuria even when he purposely consumed a large excess of carbohydrate in various forms, including starch, dextrose, and cane sugar. Except for the bother of having to be continually drinking and as continually emptying his bladder, the patient's general state did not seem to be in any way abnormal.

*Case 4.*—A woman, aged thirty-two, was the owner and manager of a farm. She was in perfectly good health until, half way through one of her pregnancies, a flood occurred, destroying all her crops and rendering her almost penniless. This sudden and severe shock was immediately followed by typical diabetes insipidus, with the severe polydipsia and polyuria already described. She far exceeded all the other three cases we have described in the extent of her polyuria. She once passed as much as five gallons of urine in twenty-four hours, and she persistently passed gallons rather than pints.

Her general health, however, remained fairly good, and not only did she survive the pregnancy during which the diabetes insipidus set in, but she actually became pregnant again and went to full term. Delivery was normal. During the pregnancy the polyuria abated somewhat, but the least quantity of urine passed in one day was even then nine pints. After the birth of the baby the urine at once increased in quantity again though, except for her thirst and her frequency of micturition, she was in perfect health when she left the hospital. Some time afterwards, however, she was attacked by double lobar pneumonia, and from this she died.

